# A Lecture on the Modern Improvements in Surgery

**Published:** 1845-06

**Authors:** John Houston


*A Lecture on the Modern Improvements in Surgery.*—(Delivered 4th November, 1844.) Being Introductory to a Course of Lectures on Surgery in the School of Medicine, Park-street, Dublin. By John Houston, Esq., M. D. M. R. I. A. Apology—Chemistry and Histology—The blood—Inflammation—Union by the first intention—Military surgery—Amputation—Lower jaw—Joints—Subcutaneous sections—Autoplasty—Aneurism—Nævus—Tracheotomy—Hydrothorax—Tympanitis—Hydrocephalus—Fractures—Artificial anus—Urinary diseases—Lithotomy and Lithotrity—Rectum—Syphilis.

Gentlemen,—For success in any of the pursuits of life, a proper estimate of the importance of the object aimed at, and of the extent of the means to be used for its attainment are the first requisites. To no pursuit does this observation apply more forcibly than to that of medicine—a science which, by some strange perversion of fact, is regarded by men, generally, as one easy of attainment, but one which, nevertheless, as the practitioner finds, often when too late for remedy, requires much patient toil and hard-earned experience, and which, even at the end of a long and laborious life, is often left still unlearned. To incûlcate, then, upon such of you as are beginners, as well as to remind those who have already made some progress in study, the extent and importance of the profession in which you are embarked, and to incite you to the necessary energy in its prosecution, I have thought of the plan, which I shall now proceed to carry out, of bringing before you some of the most striking improvements which have taken place in latter years, confining myself to the topics which legitimately belong to my own department of surgery, and the details of which I shall have the opportunity of filling up in the progress of the course hereafter. A lesson of this kind, by exhibiting to view the pursuits and energies of the great men of our profession, and the path to fame which still lies open to new aspirants, may, I hope, make a useful and lasting impression on the plastic and ardent minds of at least, some of my youthful hearers.

To render fully intelligible the modern improvements in surgery, it would be necessary to begin by demonstrating the modern improvements in anatomy and physiology, on which, especially, many of the former are founded; but whilst I cannot here stop to do more than allude to one or two, in illustration of the bearing of such investigations on practical surgery, I may take the opportunity of apprizing you that there never was a period in the history of the profession in which an exact knowledge of the structure and functions of the body—a knowledge to be obtained only by the combined aid of chemistry and histology—bore so directiy on medical theory and medical practice. The retort and the microscope are now becoming as much the pocket companions of the practitioner as the lancet and the stethoscope; and it is curious to observe how the up-hill tracks of the stethoscope to public favor, in former days, have shadowed out that through which the microscope is now destined to wind its tardy way.

Recent researches in the physiology of the blood, in connection with the process of inflammation—that morbid condition which bears so important a part in all organic lesions—have thrown much light on disease.

The great mind of John Hunter saw and believed that the blood possessed in itself an independent life, even while circulating loosely in the bloodvessels, but he knew not the nature and seat of that vitality. The discovery was reserved for the physiologists of our days. There are particles termed globules floating in this liquid, about the 3000th part of an inch in diameter, or so small that myriads of them are contained in a single drop. It has been ascertained, respecting these globules, that they are, each and all, endowed with a definite and uniform shape, and with a development, in virtue of which they pass, by successive transitions, from a condition of origin to one of final evolution—a veritable organization, in other words—properties which give them a claim to the title of life as much as those which justify the application of that term to the ovum from which proud man himself dates his being. The atomic particles of which the blood is composed being thus individually alive, collectively, they form a mass, of which it may literally, as well as allegorically, be said, "For it is the life of all flesh, the blood of it is for the life thereof." For the life of the flesh is in the blood.

But there is still another discovery which has been of late made, and which promises to be of great value in organic physiology—viz., that there are at least two kinds of globules in the blood, one yellow or red, the other transparent or white, and which differ from each other essentially, both in form and organization. The uses of these particles respectively, although involving some great and important functions in the animal economy, are not yet known with sufficient exactitude to be enunciated as ascertained facts in physiology. I may state, however, as a prevailing opinion, that the red globules, which consist each of a vesicle holding a ferruginous fluid in its interior, are the receptacles and the carriers of the oxygen which is destined to kindle and maintain the excitability of the different organs of the body, in the way that the oxygen of the atmosphere, propelled upon heated coals, raises and keeps up a flame; whilst the white globules are exclusively and essentially nucleated cells, floating stores of living elements derived from the food eaten—the true pabulum vitæ destined for the nutrition and growth of the frame. In furtherance of this latter view, Mr. Addison, of Malvern, has instituted experiments which show that an animal structure, bearing all the chracters of cellular or rather fibrous tissue, may be formed, synthetically, out of the contents of these cells, by the addition of an alkali, and he ventures on the moral position that some analogous amalgam may take place in the growth of animal tissues from the blood, both in health and disease. Now, without offering any opinion on that part of the theory of Mr. Addison which bears reference to the exclusiveness of the white globules in this office, I may state that I concur in his statements respecting the fibrillation of their contents by the action of a dilute alkali, and their fecundity in the production of granules. And I will even go farther with him and say, that those granules are themselves, each and all, possessed of an independent life. I have repeatedly watched them, and have shown them to others, when burst from their cell-membrane, performing sundry independent, and apparently voluntary, evolutions in the field of the microscope, until, to the eye, the whole looked like a moving mass of creeping things. In this view, then, the blood is doubly alive, as exhibited—first, in its forming and taking part in the repairs of the animal machine, and, secondly, in the independent movements possessed by the ultimate particles of its matter.

The red globules are small and pliable, and glide with a facility which partakes almost of a repulsion, through the fine capillary tubes of the vessels, whilst the white ones, of a size nearly one-third larger, round and determinate in form, lag slowly along, as if influenced by a sort of prospective attraction to their walls. You can observe, in the vessels of the web of the frog, under the microscope, this double current—viz., one of red particles running in the centre of the stream, and another of white ones, stealing along the sides of the vessels; and if in the frog, so equally in man, the only difference between them in this respect being, that in the former, where the vessels are transparent, the blood is to be seen by the eye, whilst in the latter, in which they are opaque, the fluid is hidden from the sight.

Such being, incontestably, the manner of the circulation in health; let us enquire what it becomes during inflammation. Under inflammation, the tendency of the white globules to linger in the part is increased. They accumulate in the capillaries, and so stop up the stream that the mass of the blood actually stagnates there; and hence the origin and final manifestation of the *rubor*, *tumor*, *calor*, *cum dolore* of an inflamed spot. These magnified diagrams of the circulation in the web of the frog's foot, taken from Dr. Hughes Bennett's new "Treatise on Inflammation," Dr. William's "Principles of Medicine," and Mr. Addison's "Experimental Researches," and which, particularly the first, must be attentively studied, will enable you to comprehend readily all that I can here stay to inform you of.

Whether this stoppage be the consequence of the white globules sticking mechanically in the capillaries, in such a way that they cannot get in further; or whether it arise from a want of tone in the bloodvessels increasing the disposition to attraction between them and the globules—which of these, I say, is the true explanation, is a point that at present engages the attention of pathologists. Be this as it may, however, the fact, and it is a very remarkable one, is as I have stated. The part being thus laden with an over abundance of the organizable materials of the blood, its condition becomes somewhat analogous to one of excess of nutrition, and as such the state of inflammation is regarded by some pathologists. The process of adhesion in soft parts; the development of bone in connexion with bone, of muscle with muscle, &c., as the result of the imflammatory state, are all easily understood under this view.

In respect of union by the first intention—a process of great importance in reference to surgical operations—we are no longer puzzled and perplexed, by vague and fanciful notions, about vessels uniting mouth to mouth, or shooting their tendril-like tubes from one side of a wound to another. The ancients were perfectly acquainted with the fact of such inosculations, but they knew not the *modus operandi* of the process. A beautiful experiment, instituted by Troja, shows how alive was their attention to this subject. He cut across the leg of a fowl by three sections, in such a manner and at such intervals as to allow one section to be well healed before he made another, but so completely, on the whole, that no part remained which had not been divided. He then killed the fowl, and threw an injection into its blood vessels, when he found that the injection had passed as fully into the toes of the amputated as the unamputated limb, proving, of course, that it had found a way through the vessels formed *de nova* in the recently united textures. But Troja knew then no more of the matter than that the fact was so. Recent and valuable discoveries have, however, filled up this hiatus in pathology, and we now know that it is by a process of cell-genesis that the act is accomplished—viz., that the nucleated cells generated in the lymph exuded at the wound, and which serves as a sort of plasma or manure for their propagation, arranging themselves into all sorts of appropriate and necessary forms, are converted into textures analogous, in every respect, to those from whose neighborhood they have sprung; forming thus in one place, bloodvessels, in the very walls of which, as proving their cell-origin, the nuclei of the cells are discernible, lying before the eye, like pieces of wood pavement, at definite given distances, and that even long after the growth of the vessels is complete; forming, in another place, nerves; in another, cellular tissue; and so on, until a complete re-establishment of the living organized medium is effected. These newly discovered facts, in connection with others to which they naturally lead, bearing on the maintenance of an healthy condition of the cells, as well as of the plasma in which they are to be developed, must have a most important influence both on the theory and practice of surgery.

For the present improved system of treatment of many surgical affections, much is due to the *surgeons of the army.* It was in the field of battle that a practice which had existed for ages—that of dipping the stumps of amputated limbs into boiling pitch or oil, to stop the hemorrhage—was first discontinued. On one occasion, after a great slaughter in battle, and when his stock of boiling oil had run out, Ambrose Parè was obliged to leave the stumps of many of the wounded to what he considered the more unprofessional plan of wrapping them round in wet clothes, and in the expectation of finding, also, such dead by the next day. Matters turned out quite otherwise, however; for whilst those treated by the oil had been, of course, in pain, sleepless and feverish, those whose wounds were dressed simply, had enjoyed ease and sleep, and lay comparatively cool.

From this incident, the resources of the constitution in arresting hemorrhage began to be understood, and a more humane and judicious practice to be adopted. The reputation of the surgeon, at the time, became universal. The soldiers disregarded danger whenever he was present. On an occasion in which Metz was besieged, and the wounded were dying without medical aid, Parè was brought into the city. The soldiers, when apprised of his arrival, cried out, "Our Parè is with us! we have nothing to fear!" and then fought to conquer. The late Continental wars found an Ambrose Parè in every regiment. Our own Hennen and Guthrie, and S. Cooper and Ballingall, and the great Baron Larrey of Napoleon, have transmitted to us the records of the discoveries and improvements, as well as of the humanity and bravery, of their respective soldier surgeons. Civilians, even, have been found zealous and courageous enough to quit the safe retirement and practice of the domestic circle for such scenes of carnage and of danger, in order to be able to bring back to us intelligence regarding the nature and treatment of wounds, as noticed in such wide fields of observation. John Hunter, John Thompson, and Sir Charles Bell, have immortalized themselves by their devotion and services on this head.

In regard of *amputations*, the greatest modern improvement is, the frequency with which they are abstained from. When surgeons first got into the way of operating, limbs were removed without scruple, and, frequently, without just cause.

They would appear sometimes to have been lopped off, as if to prove how well the body could maintain its existence without them. Morand relates that in the Hôtel des Invalides, at Paris, mutilated objects are in recollection, who had lost their thighs and arms, so that, unless assisted, they could not stir, and it was necesary to feed and wait upon them like new-born infants. That such a state of things has long passed away is quite true, even within our own time still further improvements in this respect have been made, and many limbs are now saved, that, not long since would, to a certainty have been condemned to the knife. Sir Benjamin Brodie informs us, in the last edition of his work on the "Diseases of the Joints," that it was the practice which prevailed in his early days, of amputating white swellings, as soon as their character as such was determined, that gave him those opportunities of investigating the disease in its early stages, on the pathological facts derived from which the chief value of his book depends. Our museums in this city, likewise, bear evidence to the same practice of early amputation; and those who possess such preparations of disease will do well to take care of them, as they are not likely to get many other similar specimens from the hand of modern surgery.

The same observations apply equally to many other cases, such as diseases of the mamma and of the testis, ulcers of the legs, hernia, injuries of the head, compound fractures and dislocations, &c., all of which yield, oftentimes, to improved plans of treatment, short of having recourse to the knife. Regarding hernia, the name of O'Beirne will be hereafter associated with it, as having introduced a plan calculated to save many a valuable life. I allude to his method of drawing the gas from the interior of the bowel by means of a long gum-elastic tube; and to the efficacy of which there is now abundant evidence from all quarters.

In respect of operations, then, true surgery rather avoids than courts them; and, in this respect, unlike what takes place in all other professions, the improvements introduced into it cause a dimunition in the emoluments derivable from the practice of surgery. It is a well established fact, that the incomes of medical men are much reduced from this cause, and yet, nevertheless, they persevere with laudable disinterestedness in their endeavours to effect still further improvements. Is not this the highest degree of philanthropy? In this city, more perhaps than in any other in the world, isthis statement—regarding the avoidence of unnecessary operations true. I do nothold out to you, therefore, the prospect of the exhibition of numerous surgical operations: on the contrary, I promise to you that, whatsoever hospital you may select for attendance, you will there see every judicious effort made at cure, by remedial means, before having recourse to the knife. The principle that you will see acted upon is, that to preserve a limb is better than to cut it off.

The diminution of surgical operations in this city is our highest boast; and I make it thus publicly, to contrast with one of an opposite character, which I have heard of as having been uttered elsewhere, for the ignoble purpose of attracting students to the schools. And I do so, still more especially, because I find that the records of the hospitals of Dublin have been pried into, in order to make a case for the assertion of such a discreditable comparison. But although, in one sense, there is a judicious diminution in the number of surgical operations, in another, there is an increase. Many operations, unknown in former days, are now in common practice.

The grand exploit of amputating the *lower jaw*, even from its articulations, the boldness of which has been only equalled by its success, has now become a standard operation in surgery. Persons afflicted with the distressing and loathsome disease [showing a drawing of it], for which this operation is undertaken, were formerly allowed to die without any idea being entertained of the possibility of saving them; but now that a great mind, relying on a sound knowledge of the capabilities of the human frame, has set the example of extirpating the diseased mass *in toto*, many surgeons have fearlessly followed in the path thus laid open for them, and have derived honor from the success which crowned the enterprise. The success of this opeation—both as regards immunity from danger, rapidity of convalescence, and the useful quality of masticatory apparatus which follows—is almost incredible. Mr. Cussack has operated twelve times, and here [showing them] are the preparations, casts, and drawings of the whole series. Now, in all these cases there has been but one death, and that, not as the result of the operation, but from the erysipelas. I was present, a few weeks ago, at one of these operations, in which he removed one half the jaw, including the articulation on one side. The operation was performed on a Wednesday, and on the Monday following, when I visited the patient, I found the great external incision united from end to end by the first intention, and the patient himself setting up by the fire and in the act of finishing a long letter to his friends. Before the end of the third week he was quite well, and had traveled home to the country—a distance of upwards of one hundred miles. It is quite certain, too, that after such operations, when half and even more than half, of the jaw bone is removed, the individuals, nevertheless, retain good powers of mastication and of speech. And shall I not call this a *modern* improvement in surgery, when the great author and champion of it is seated amongst us in this room?

There is an improvement, of somewhat the same kind, and one for the introduction of which into practice, Sir Philip Crampton, of this city, and Mr. Syme, of Edingburgh, share the honor—viz., the removal, in diseases of certain *joints*, of the unsound parts only of the bones. In affections of the elbow-joint this may be obviously a great improvement, as the preservation of the hand is a most desirable object in every case. But, in regard of the knee, neither the value nor propriety of the operation is so striking. On the whole, however, it is a bold and important innovation on the old practice of cutting off an entire limb on account of disease in a part only; and reflects honor on the distinguished gentlemen who have concieved and carried it into execution.

Of all the modern improvements in surgery, none are more curious, and few more important, than those of the kind that are called operations by the *subscutaneous section*—operations which consist in dividing deep parts, more especially tendinous and muscular structures, from within as it were, or, in a manner, to exclude the part thus wounded from any communication with the external atmosphere. Examples of its application are seen in the new and successful methods of removing the deformities of club-foot, wry neck, &c. Surgeons, influenced, perhaps, in some degree, by the aphorism of Hippocrates, that "wounds of tendinous parts are mortal," were, until lately, afraid to meddle with such textures. Even so recently as the days of my own pupilage, warnings on this head were reiterated, year after year, in public lectures. Inflammation of synovial membranes; erysipelas, and lock-jaw, were the quicksands, held out as besetting the application of the knife to such textures. But now such dangers are only looked upon as chimerical. Five hundred operations by the subscutaneous section have been lately counted as having been performed without the occurrence of a single instance of bad consequence. The first attempt at an operation of this kind was made in Holland, in the year 1685, by Isacius Minius, but it was badly done, and, as it failed, was not repeated. The second was at Montpelier, in 1816, by Delpech, and although a certain amount of success accompanied the attempt the operation was not followed up, and it became a dead letter. The third epoch in the history of the operation is 1831, when it was taken up by Stromeyer, in Germany, who practised, and advocated it so successfully, that it has found favor in all parts of the world, and has now become a favorite and fashionable operation. The discovery of a new principle in medical science is soon found to diffuse its benefits in various directions, and so it has here. The practice, which at first was adopted solely in reference to division of tendons in deformed limbs, is now applied to the relief or cure of many other ailments. It has been employed by my friend and colleague in the City of Dublin Hospital, Mr. Williams, for chronic thickening, with enlargement and distention of bursal sacs, such as that in front of the knee in charwomen, and for which, previously there was no known remedy but excision. This operation I have myself performed on several occasions with success. The practice of scarifying the ends of bones in ununited fractures, by the subcutaneous section, for the purpose of promoting the secretion of callus, is another new application of the principle. Deformities from burns and scalds sometimes admit of remedy by subcutaneous division of the contraction. And curvatures of the spine are said to have been removed by extensive hidden incision of the muscles of the back. M. Guerin tells us that he divided fifty muscles in a hump-back in this way, and not only was there success after the operation, but also an absence of all pain or local inflammation in the parts so divided. I mention this, however, not so much in laudation of this mode of treatment in such cases, as in illustration of the principle and the fact, that extensive deep incisions give rise, if unaccompanied by exposure to the air, to comparatively little local disturbance.

You have all heard of the operation for the cure of squint. This operation suggested itself out of the discoveries of Stromeyer, and deservedly holds a place among the modern improvements in surgery. But it is principally in its application to club-foot that such beneficial and unexpected results have flowed out of this discovery. This sad deformity, than which none other to which the frame is liable so thoroughly embitters life, or disqualifies for the taking a part in the common affairs of society, and which had, hitherto, set all attempts at remedy at defiance, now yields to the improved system, and is no longer the canker of domestic happiness, or the opprobrium of the medical profession. In childhood, this deformity may, with certainty, be now remedied; and, even in adult life, when the bones and the textures have grown into a confirmed mishape, it is extraordinary how nearly they may be brought back to a normal state by attention and perseverance after befitting subcutaneous sections.

Almost discrediting the first statements that reached us on this subject, I proceeded to put the matter to the test of experiment, when the results proved, to my satisfaction, the true value of the improvement. One case of the kind I may be permitted to state, by way of illustration. It is that of a fine young woman with talipes varus, the cast of whose foot is here exhibited. She was nineteen years of age, handsome and intelligent, but exhibiting that timid and subdued mien which the consciousness of such a defect always gives to the character. The deformity was so immovably fixed, that I found it necessary to divide even a greater number of unyielding parts than common, as in addition to several tendons of muscles and other fibrous textures on the inside of the foot, I had also to cut across, completely, the broad plantar fascia. In three months, the foot presented, and retained, when stripped of the straightening apparatus, this perfect form, [exhibiting the cast]. In six months she could stand on it, laying the sole to the ground with tolerable firmness; and in eighteen months she walked steadily and well, and had already got several sweethearts in her train: which circumstance I mention as being, perhaps, one of the best proofs I could offer of her having acquired a neat foot and ankle, and as one, certainly, of the chief sources of her gratitude to me.

It is impossible to conceive the amount of good which in future times may accrue to society at large, from the carrying out of such an improvement; but we are, nevertheless, in a condition, in some measure, to estimate and to grieve for the losses which the world has sustained by even the tardiness of its introduction. Had this operation been known in the youthful days of Lord Byron, and applied to the relief of that nobleman's deformity, a genius which was soured by the consciousness of a personal defect, and which received therefrom a misanthropic bias, might have blossomed in the sunshine of generous boyhood, and, when mature, have brought forth manna, instead of gall and wormwood.

[TO BE CONTINUED.]

